# Comparison of the predictive capability of antral follicle count vs. the anti-Müllerian hormone for ovarian response in infertile women

**DOI:** 10.3389/fendo.2022.862733

**Published:** 2022-10-31

**Authors:** Xingyu Sun, Wang Xiong, Liting Liu, Junjun Xiong, Chenlu Liao, Yunzhu Lan, Feifei Li, Shufei Tao, Muzi Meng, Chenyu Sun, Xiguang Mao

**Affiliations:** ^1^ Department of Gynecology, The Affiliated Traditional Chinese Medicine Hospital of Southwest Medical University, Luzhou, China; ^2^ Department of Gynecology, The Affiliated Hospital of Southwest Medical University, Luzhou, China; ^3^ Department of Ophthalmology, The First People’s Hospital of Neijiang, Neijiang, China; ^4^ Family Medicine, Carle Foundation Hospital, Urbana, IL, United States; ^5^ American University of the Caribbean School of Medicine, Preston, United Kingdom; ^6^ Bronxcare Health System, The Bronx, NY, United States; ^7^ AMITA Health Saint Joseph Hospital Chicago, Chicago, IL, United States

**Keywords:** Anti-Mullerian hormone, Antral follicle count, Female infertility, *In vitro* fertilization-embryo transfer, Ovarian response

## Abstract

**Objectives:**

The aim of this study was to compare the predictive capability of antral follicle count (AFC) and the anti-Müllerian hormone (AMH) on ovarian response in infertile women and to identify potential factors influencing retrieved oocytes.

**Methods:**

A total of 2585 infertile women who underwent *in vitro* fertilization/intracytoplasmic sperm injection (IVF/ICSI) cycles had been enrolled in this study. Spearman correlation was used to investigate the correlation between retrieved oocytes and AFC. Multiple linear regression analysis was used to study the parameters affecting the number of retrieved oocytes.

**Results:**

Spearman correlation and multiple linear regression analysis revealed that the oocyte retrieval number was positively correlated with AFC (*r* = 0.651, p < 0.001) and AMH (*r* = 0.566, p < 0.001) and negatively correlated with age (*r* = -0.425, p < 0.001) and regimen selection (*r* = -0.233 p < 0.001). There was no significant correlation between retrieved oocytes and BMI (p = 0.913). ROC analysis revealed that AFC was a better predictor of adverse effects than AMH, BMI, and age (AUC: 0.916 VS 0.791, 0.575, 0.752). Meanwhile, AFC and AMH were comparable in predicting high response (AUC = 0.731 and AUC = 0.733, respectively).

**Conclusions:**

This study showed that retrieved oocytes were positively correlated with serum AMH and AFC and negatively correlated with age and BMI. AFC had an ideal predictive performance in ovarian response prediction. The mechanism of the effect of AFC on ovarian response during controlled ovarian hyperstimulation (COH) needs to be further investigated.

## Introduction

The term ovarian response to controlled ovarian hyperstimulation (COH) refers to women undergoing *in vitro* fertilization and embryo transfer to exogenous gonadotropin stimulation (1). COH is an important component of *in vitro* fertilization and embryo transfer (IVF-ET) technology. Different COH protocols can elicit different ovarian responses in the same patient. Meanwhile, the ovarian response to COH is an important factor affecting the clinical pregnancy outcome in patients, and different ovarian responses can have different effects on pregnancy (2). However, there is still a lack of validated biomarkers to predict ovarian response.

Anti-Müllerian hormone (AMH) is considered a member of the transforming growth factor B family (3) and has been shown in recent years to exist as a dimeric glycoprotein. Serum AMH levels are closely related to the number of antral follicles and can be a valuable parameter for predicting ovarian response and pregnancy in assisted reproductive technology (ART) cycles (4–6). Serum AMH levels vary with follicle-stimulating hormone (FSH) levels and may remain stable throughout the menstrual cycle. The absence of changes in serum AMH levels may be related to the continued development of small follicles, which have the potential to grow independently of cyclic changes. Therefore, AMH can be a valuable and intuitive indicator (7–11).

In this study, the data had been collected from patients who were treated with *in vitro* fertilization/intracytoplasmic sperm injection (IVF/ICSI) at the reproductive center in our hospital. The relationship between serum AMH level, the ovarian antral follicle count (AFC), body mass index (BMI), age and other factors, and ovarian response was analyzed. The knowledge gained from the study can better guide clinicians in choosing the most appropriate ovulation promotion regimen, therefore improving the pregnancy rate in infertile women.

## Methods

### Patient enrollment

This was a retrospective study of data from infertile women who underwent the first cycle of *in vitro* fertilization/intracytoplasmic sperm injection (IVF/ICSI) cycles at the reproductive center of the affiliated hospital of Southwest Medical University (Luzhou, China) from October 2018 to September 2020.

Inclusion criteria were as follows:

1. Infertile women treated with IVF-ET or ICSI-ET treatment at the reproductive center;
2. Use of different protocols;
3. The presence of bilateral ovaries confirmed by color Doppler ultrasonography.

Exclusion criteria were as follows:

1. Ovarian surgery or ovarian lesions;
2. Ultrasonography showing unclear and absent ovaries;
3. Systemic endocrine diseases such as polycystic ovary syndrome, hyperprolactinemia, etc.
4. Severe endometriosis.

### Data collection

Data collected included age, BMI, AMH, AFC, Gn medication days, Gn dosage, E_2_ on the hCG triggering day, retrieved oocytes, available embryos, etc.

### AMH test

Blood samples from all patients were stored at -20°C and tested using an enzyme-linked immunosorbent assay kit (AMH quantitative kit, Guangzhou Kangrun Biotechnology Co., Ltd.). The minimum detectable AMH concentration was 0.06 ng/ml; the correlation coefficient, *r* ≥ 0.9900; the relative deviation of the assay results, within 10%; and the coefficient of variation (CV), ≤10%.

### AFC measurement

The antral follicle count (AFC) numbers were counted using the Color Doppler ultrasonic diagnostic apparatus (LOGIQ-400BW model, GE, USA) on day 2.

### Controlled ovarian hyperstimulation

The ovarian stimulation program was selected according to the patient’s age, body mass index (BMI), basal endocrine level, and the number of antral follicles.

### The definition of ovarian response

In this study, patients were divided into groups according to ovarian response. Low ovarian response group: number of eggs retrieved ≤3 or cancelled due to low ovarian response; Normal ovarian response group: 3< number of eggs retrieved ≤15; High ovarian response group: number of eggs retrieved >15.

### The treatment protocols

Depending on the basal secretion of the patients, the follicle-stimulating hormone (FSH) (Gonal-F, EMD Serono, Rockland, MA, USA) combined with the gonadotropin-releasing hormone (GnRH) agonist (Decapeptyl; Ferring, Kiel, Germany) and antagonist (Cetrotide; Merck Serono, Darmstadt, Germany), was mainly used in controlled ovarian hyperstimulation proposals for ovarian stimulation. Controlled ovarian hyperstimulation protocols include GnRH agonist long protocols, GnRH agonist short protocols, GnRH antagonist protocols, and luteal-phase ovarian stimulation protocols. The choice of FSH injection dose is based on the women’s ages, ovarian reserve, and various reactions. Follicular development was monitored by transvaginal ultrasound. Therefore, 10,000 U of recombinant human chorionic gonadotropin (r-hCG) (Ovidrel; Merck Serono, Darmstadt, Germany) was used to induce ovulation 36 h before puncture egg retrieval when the dominant follicle grew to a diameter of 18 mm or more, or when the average diameter of the three dominant follicles reached 17 mm.

### Statistical analysis

Statistical analyses were performed using the SPSS statistical software (v. 23, IBM Crop, Armonk, NY, USA). The Kolmogorov–Smirnov test was used to determine whether the measures showed a normal distribution. Data not conforming to a normal distribution were expressed as median (25th–75th percentiles), and the Kruskal-Wallis test was used because the distribution of the parameters was non-normal. Count data were expressed as percentages (%), and when the proportions were very small (<1.0%), Fisher’s exact test was used for comparison. Multiple stepwise linear regression analysis was used to analyze the factors affecting the oocyte recovery rate. Spearman’s correlation coefficient was applied for correlation analysis. The area under the ROC curves (AUCs) was calculated to analyze the relationship between the retrieved oocytes, AFC, age, BMI, and AMH. The Youden index was calculated, and the maximum value of the Youden index was used as the cut-off value. The predictive value was determined using the Youden index, and a p-value less than 0.05 (p  <  0.05) indicated a statistically significant analysis.

## Results

### Basic clinical information of all patients in different groups

A total of 2,585 patients undergoing their first cycle of *in vitro* fertilization and embryo transfer or intracytoplasmic sperm injection and embryo transfer treatment (IVF-ET/ICSI-ET) were included in this study. Patients were divided into three groups according to ovarian response (low ovarian response, n = 441; normal ovarian response, n = 1,738; high ovarian response, n = 406). Baseline characteristics are shown in **Table 1**. Our findings showed that the mean age and BMI of the patients gradually decreased, and the average AMH level gradually increased from the low ovarian response group to the normal ovarian response group and from the normal ovarian response group to the high ovarian response group. The difference between the three groups was statistically significant (p < 0.05). The detailed protocols used by the patients were as follows: 34 natural cycles, 212 short protocols, 998 long protocols, 1097 antagonist protocols, 230 PPOS protocols, and 14 mild stimulation protocols. PPOS protocol was used mainly in the low ovarian response group. The antagonist protocol was mainly used in the normal ovarian response group, and the long protocol was used in the normal ovarian response group.

**Table 1:** Comparison of conditions in different ovarian response groups.

	Low ovarian responsen = 441	Normal ovarian responsen = 1738	High ovarian responsen = 406	p-Value
Age (years)	37 (33-42)	31 (28-34)	29 (26-31)	p < 0.05
BMI (kg/m^2^)	22.94 (20.89-24.98)	22.03▲ (20.03-24.24)	21.79▲ (20.03-23.87)	p < 0.05#
AMH (µg/l)	1.11 (0.47-1.80)	2.68 (1.42-4.68)	5.4 (3.14-8.31)	p < 0.05#
Natural cycles (%)	31 (7)	3 (0.2)	0 (0)	p < 0.05#
Short protocols (%)	74 (16.8)	133 (7.6)	5 (1.2)	p < 0.05#
Long protocols (%)	29 (7)	701 (40.3)	268 (66)	p < 0.05#
Antagonist protocols (%)	147 (33.3)	818 (47.1)	132 (32.5)	p < 0.05#
PPOS (%)	149 (33.8)	80 (4.6)	1 (0.2)	p < 0.05#
Mild stimulation protocols (%)	11 (2.5)	3 (0.2)	0 (0)	p < 0.05#
AFC (pieces)	5 (4-7)	12 (8-16)	16 (14-17)	p < 0.05#
Gn medication days (d)	8 (7-9)	10 (9-12)	11 (10-12)	p < 0.05#
Gn dosage (U)	2475 (1800-3225)★	2550 (2025-3000)★	1993.75 (1421.88-2625)	p < 0.05
E2 on the hCG triggering day (pmol/l)	726.39 (376.48–1141.41)	2399.85 (1832.54–3307.17)	4421 (4184.74–5507.15)	p < 0.05
Retrieved oocytes (pieces)	2 (1–3)	9 (6–12)	18 (17–19)	p < 0.05
Available embryos (pieces)	1 (0–1)	4 (2–5)	7 (5–9)	p < 0.05

LR, low ovarian response; NR, normal ovarian response; HR, high ovarian response.

BMI, body mass index, BMI = weight (kg)/height (m^2^); AMH, anti-Müllerian hormone; PPOS, progestin-primed ovarian stimulation; AFC, antral follicle count;

Gn: gonadotropin.

▲Group NR and group HR have no significant differences in BMI (p > 0.05).

**★**Group LR and group NR have no significant differences in Gn dosage (p > 0.05).

The mean AFC, E_2_ on the hCG trigger day, and Gn days gradually decreased from the high ovarian response group to the normal ovarian response group and from the normal ovarian response group to the low ovarian response group. The mean total Gn dose was greatest in the normal ovarian response group and lowest in the ovarian high response group. The differences between the three groups were statistically significant (p < 0.05) for the short protocols, long protocols, PPOS protocols, AFC, Gn dosing days, Gn dosage, and hCG trigger day E2. The retrieved oocytes and available embryos decreased from the high ovarian response group to the normal ovarian response group and from the normal to the low ovarian response group. The difference between the three groups was statistically significant (p < 0.05).

### Correlation analysis between retrieved oocytes and affecting factors

**Figure 1:**
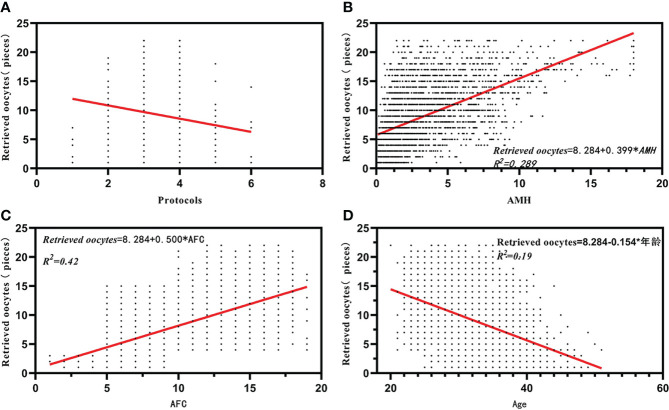
Correlation analysis between retrieved oocytes and affecting factors. **(A)** The correlation between retrieved oocytes and protocals. **(B)** The correlation between retrieved oocytes and AMH. **(C)** The correlation between retrieved oocytes and AFC. **(D)** The correlation between retrieved oocytes and Age.

Stepwise multiple linear regression analysis revealed that AFC and AMH were positively correlated with oocyte retrieval (*B* = 0.500 and *B* = 0.399, p = 0.000 and p = 0.000). Similarly, stepwise multiple linear regression analysis also showed that age and treatment regimen were negatively associated with oocyte retrieval (*B* = –0.154 and *B* = –0.355, p = 0.000 and p = 0.000, respectively). There was no significant correlation between retrieved oocytes and BMI (p = 0.913) (**Table 2**). Correlation analysis using Spearman’s correlation analysis showed a positive correlation between retrieved oocytes and AMH and AFC (*r* = 0.566, p < 0.001; *r* = 0.651, p < 0.001) (**Table 3**, **Figures 1B, C**), while the retrieved oocytes were negatively correlated with age and protocols (*r* = -0.425, p < 0.001;*r* = -0.233, p < 0.001) (**Table 3**) (**Figures 1A, D**).

**Table 2:** Analysis of the factors affecting the number of retrieved oocytes by multivariate-linear regression (stepwise method).

	*B*	*t*	p	95%CI
**Constant**	8.284	11.419	<0.001	6.862~9.707
AFC	0.500	22.875	<0.001	0.458~0.543
AMH	0.399	0.032	<0.001	0.336~0.463
Age	-0.154	-9.424	<0.001	-0.186 ~ -0.122
Protocols*	-0.355	-3.785	<0.001	-0.539 ~ -0.171
BMI	0.000	-0.110	0.913	-0.004 ~ -0.003

AFC, antral follicle count; AMH, anti-Müllerian hormone; BMI, body mass index; BMI = weight (kg)/height (m^2^); Protocols*: They were, in order, Natural cycles, Short protocols, Long protocols, Antagonist protocols, PPOS, and Mild stimulation protocols.

**Table 3:** The correlation between the number of retrieved oocytes and different influence factors by Spearman analysis.

	AFC	AMH	Age	Protocols	BMI
***r***	0.651	0.566	-0.425	-0.233	-0.086
**p**	<0.001	<0.001	<0.001	<0.001	<0.001

AFC, antral follicle count; AMH, anti-Müllerian hormone; BMI, body mass index, BMI = weight (kg)/height (m^2^); Protocols*, Natural cycles, Short protocols, Long protocols, Antagonist protocols, PPOS, and Mild stimulation protocols.

### Comparison of the prediction performance of retrieved oocytes vs. AFC, AMH, BMI, and age in the low ovarian response group

**Figure 2:**
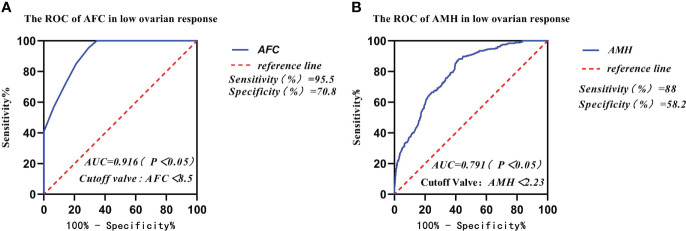
Comparison of prediction performance of Retrieved oocytes vs AFC, AMH, BMI, and age in the group of low ovarian response. **(A)** The ROC of AFC in low ovarian response; **(B)** The ROC of AMH in low ovarian response.

The receiver operating curve (ROC) was used to analyze the predictive ability of AFC, AMH, BMI, and age in infertile women with low ovarian response (**Table 4**). The area under the ROC curves (AUC) for AFC, AMH, BMI, and age was statistically significant (p < 0.05). The AUC for AFC and AMH was significantly higher than that for BMI and age (p < 0.05). The Youden Index (Youden Index = sensitivity + specificity - 1) for AMH was 0.462, with a critical value of 2.23 μg/l, sensitivity of 88%, and specificity of 58.2% (**Figure 2B**). Meanwhile, the Youden Index of AFC was 0.663, with a critical value of 8.5, sensitivity of 95.5%, and specificity of 70.8% (**Table 4**) (**Figure 2A**).

**Table 4:** Predictive comparison of different influence factors and low ovarian response.

	AUC	Cut-off Valve	Sensitivity(%)	Specificity(%)	Youden Index
***AFC***	0.916	8.5个	95.5	70.8	0.663
***AMH***	0.791	2.23	88	58.2	0.462
***BMI***	0.575	22.59	56.2	57	0.132
***Age***	0.752	33.50	69.4	69.3	0.387

AFC, antral follicle count; AMH, anti-Müllerian hormone; BMI, body mass index, BMI = weight (kg)/height (m^2^).

### Comparison of the prediction performance of retrieved oocytes vs. AFC, AMH, BMI, and age in the high ovarian response group

**Figure 3:**
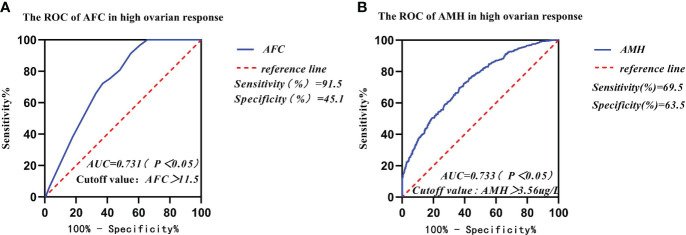
Comparison of prediction performance of Retrieved oocytes vs AFC, AMH, BMI, and age in the group of high ovarian response. **(A)** The ROC of AFC in high ovarian response; **(B)** The ROC of AMH in high ovarian response.

The AUCs of AFC, AMH, BMI, and age in infertile women with high ovarian response were 0.731, 0.733, 0.511, and 0.654, respectively, as analyzed by the subject ROC (**Table 5**). The AUCs of AFC and AMH were significantly higher than those of BMI and age (p < 0.05). The Youden Index (Youden Index = sensitivity + specificity - 1) for AFC was 0.364, with a critical value of 11.5, sensitivity of 91.4%, and specificity of 45.1% (**Figure 3A**). Meanwhile, the Youden Index of AMH was 0.329, with a critical value of 3.56 μg/l, sensitivity of 69.5%, and specificity of 63.5% (**Figure 3B**).

**Table 5:** Predictive comparison of different influence factors and high ovarian response.

	AUC	Cut-off Valve	Sensitivity(%)	Specificity(%)	Youden Index
***AFC***	0.731	11.5	91.4	45.1	0.364
***AMH***	0.733	3.56	69.5	63.5	0.329
***BMI***	0.511	23.8	74.4	0.3	0.044
***Age***	0.654	30.5	0.672	0.562	0.235

AFC, antral follicle count; AMH, anti-Müllerian hormone; BMI, body mass index, BMI = weight (kg)/height (m^2^).

## Discussion

The number of retrieved oocytes and the quality of eggs can be improved by a correct and reasonable assessment of the ovarian response. Wise and correct evaluation (12) of ovarian response in women with ovarian stimulation can lead to poor ovarian response and ovarian hyperstimulation syndrome, reduce cycle cancellation rates, and improve *in vitro* fertilization and embryo transfer (IVF-ET) success rates. However, current predictors of ovarian response have some limitations and the accuracy is controversial (13–16). Therefore, a large sample of statistical data is needed to draw more accurate conclusions to guide assisted reproductive technology (ART).

It is generally accepted that the female reproductive function declines gradually from age 30 and rapidly after age 35 until it begins to decline in the decade before menopause, when fertility is almost lost. This decline in fertility is accompanied by the degeneration of the oocyte, which is manifested by a decrease in the number of normal, healthy, usable eggs. For patients >35 years of age, or <35 years of age but has risk factors for reduced ovarian reserve capacity such as adnexectomy or a history of previous ovarian surgery, radiotherapy, or chemotherapy, we recommend the aggressive evaluation of ovarian function to prevent a delay in eventual fertility. It is also important to assess ovarian function prior to the start of an IVF cycle.

Earlier studies have shown a positive correlation between serum anti-Müllerian hormone (AMH) levels and the number of antral follicles (17). Serum AMH levels provide a more realistic picture of the size of the primordial follicular pool (18). Meanwhile, anti-Müllerian hormone (AMH) levels are widely considered to be a good indicator for assessing ovarian reserve and response (19). In our study, the number of retrieved oocytes was significantly and positively correlated with antral follicle count (AFC) and the anti-Müllerian hormone (AMH) and negatively correlated with age and protocol selection. Meanwhile, multiple linear regression analysis showed no significant correlation between retrieved oocyte count and BMI. The results of our study are consistent with those of other studies (20). Other studies have also shown that relevant clinical indicators such as AMH and AFC can better predict the number of retrieved oocytes in infertility treatment (21, 22). Our study also found that serum AMH levels were better predictors of retrieved oocyte count than other clinical indicators in this study, which could guide clinicians in the rational use of drugs and reduce cycle cancellation rates to practice levels. The possible reason is that AMH levels are relatively stable throughout the menstrual cycle, and blood samples can be collected for testing at any time, reflecting the trend of ovarian reserve in a timely manner. Although AFC is essentially constant and does not change much throughout the menstrual cycle, ultrasound levels and the presence or absence of follicles or cysts in the ovary can still interfere with the results of AFC. Also, the AFC test is time-limited, during menstruation or within a few days of menstruation. Also, the effect of AFC, age, and protocol selection on the number of retrieved oocytes for female infertility deserves the physician’s attention.

In our study, data from 2,585 patients who underwent IVF-ET treatment in the first cycle had been collected and counted. We found that the women’s mean age and body mass index (BMI) gradually increased from the high ovarian response group to the low ovarian response group. The results of the study showed that AMH and AFC levels were significantly lower in infertile women in both the high ovarian response group and the low ovarian response group. The average number of oocytes retrieved and the average number of available embryos also gradually decreased. Currently, serum AMH levels and AFC are crucial indicators for the clinical assessment of ovarian response in reproductive medicine centers. In addition, serum AMH levels and AFC are considered the main parameters for assessing ovarian response (23, 24). A large retrospective study found that AMH and AFC are important indicators for developing individualized treatment for COH (25). Age has been reported in the literature to predict ovarian function. In general, ovarian function is negatively correlated with age. With increasing age, ovarian function gradually declines and ovarian response decreases, with significantly fewer oocytes being retrieved (26). In this study, age was not a good predictor of ovarian response. Individuals of the same age showed different ovarian responses, thus suggesting that many factors affect individual differences in ovarian response in the treatment of infertility, including genetic factors (27). Serum AMH and AFC tests in some young patients in the reproductive medicine clinic suggest decreased ovarian function. Thus, our data are consistent with previous studies indicating that age is not a good predictor for assessing ovarian response (26). The area under the receiver operating characteristic curves (AUCs) for AMH and AFC was 0.791 and 0.916, respectively, and was a predictor of the low ovarian response group in this study. The results for both AMH and AFC were significantly higher than those for BMI (AUC = 0.575) and age (AUC = 0.752). The AUCs of AFC and AMH (0.916 and 0.791, respectively) were significantly higher than those of BMI and age (0.575 and 0.752, respectively). Therefore, we concluded that BMI and Age had no diagnostic value in predicting ovarian response in the low- and high-response groups. In contrast, AMH and AFC have diagnostic value and can accurately predict ovarian response. The predicted value of serum AMH was 2.23 μg/l, and the sensitivity, specificity, and Youden index of AMH in the low ovarian response group was 88%, 58.2%, and 0.462, respectively. The predictive value of AFC was 8.5, and the sensitivity, specificity, and Youden index of AFC in the low ovarian response group was 95.5%, 70.8%, and 0.663, respectively. It was concluded that AFC was more sensitive in predicting low ovarian response. The predictive value of serum AFC was 11.5; the sensitivity, specificity, and Youden index of AFC in the high ovarian response group were 91.4%, 45.1%, and 45.1%, respectively. The predictive value of AMH was 3.56 µg/;, and the sensitivity, specificity, and Youden index of AMH in the high ovarian response group was 69.5%, 63.5%, and 0.329, respectively. Conclusively, AFC was also more sensitive in predicting high ovarian response.

Therefore, we suggest that AFC preferentially predicts ovarian response in the case of inconsistent AFC and AMH levels. Although AFC has a high predictive value, the measurement of AFC is affected by many objective and subjective factors such as the ultrasound instrument’s resolution and the ultrasonographer’s clinical skill level. In contrast to AFC, AMH levels remain relatively stable throughout the menstrual cycle and have little relationship with the menstrual cycle (28). Retrieved oocytes showed a significant positive correlation with AFC and serum AMH levels. We can speculate that AMH combined with AFC may be a more accurate predictor of ovarian response.

There are several potential contributions. First, in the current absence of validated biomarkers to predict ovarian response, we revealed the predictive power of AFC through a retrospective analysis of 2585 infertile women who underwent *in vitro* fertilization/embryo transfer. This finding could help physicians in making clinical decisions. Second, these findings will help inspire clinical researchers to conduct randomized controlled trials to further investigate the predictive power of AFC vs. AMH.

Our study was a retrospective analysis, and had several limitations. First, it was a single-center retrospective analysis with possible selection bias. Secondly, endometrial thickness and morphology, another major factor affecting pregnancy outcome, was not monitored. Live birth and miscarriage rates were not reported in this study. This is a shortcoming of this study, and follow-up studies should further improve the above indicators. The strength of this study is that we revealed the association between serum anti-Müllerian hormone levels and ovarian response in infertile women undergoing *in vitro* fertilization and embryo transfer in the economically underdeveloped Sichuan region.

## Conclusions

In this study, we found that the retrieved oocytes were positively correlated with serum AMH and AFC and negatively correlated with age and BMI. AFC was superior to AMH in predicting low response. Meanwhile, AFC and AMH were comparable in predicting high response. Randomized controlled prospective trials are warranted to confirm our findings and thus to help develop more effective treatment strategies for infertile women.

## Data availability statement

The original contributions presented in the study are included in the article/supplementary material. Further inquiries can be directed to the corresponding author.

## Ethics statement

This study was reviewed and approved by the Ethics Committee of the Affiliated Hospital of Southwest Medical University. Written informed consent was not required for this study, in accordance with the local legislation and institutional requirements.

## Author contributions

Conceptualization: XM. Data curation and formal analysis: XS, WX, LL, JX, CL, YL and FL. Writing original draft preparation: XS. Writing, Review and Editing: ST, MM, and CS. All authors contributed to the article and approved the submitted version.

## Acknowledgments

We thank the survey participants and all staff involved in this study for their painstaking efforts in conducting the data collection.

## Conflict of interest

The authors declare that the research was conducted in the absence of any commercial or financial relationships that could be construed as a potential conflict of interest.

## Publisher’s note

All claims expressed in this article are solely those of the authors and do not necessarily represent those of their affiliated organizations, or those of the publisher, the editors and the reviewers. Any product that may be evaluated in this article, or claim that may be made by its manufacturer, is not guaranteed or endorsed by the publisher.
